# Optimization of Oligosaccharide Production from *Leuconostoc lactis* Using a Response Surface Methodology and the Immunostimulating Effects of These Oligosaccharides on Macrophage Cells

**DOI:** 10.3390/molecules23092118

**Published:** 2018-08-23

**Authors:** Sulhee Lee, Gwi-Gun Park, Jae-Kweon Jang, Young-Seo Park

**Affiliations:** 1 Department of Food Science and Biotechnology, Gachon University, Seongnam 13120, Korea; sulhee2340@gmail.com (S.L.); ggpark@gachon.ac.kr (G.-G.P.); 2 Food Nutrition Major, School of Food, Chungkang College of Cultural Industries, Icheon 17390, Korea; jkjang@chungkang.ac.kr

**Keywords:** response surface methodology, central composite design, oligosaccharide, optimization, immunostimulation, RAW264.7, lactic acid bacteria

## Abstract

Production of oligosaccharides from *Leuconostoc lactis* CCK940 was optimized using a response surface methodology with a central composite design. Culture temperature and the concentrations of sucrose and maltose were used as the main factors. The predicted optimum conditions for the production of oligosaccharides were a culture temperature of 30 °C, a sucrose concentration of 9.6% (*w*/*v*), and a maltose concentration of 7.4% (*w*/*v*). Using these optimal conditions, *Leuconostoc lactis* CCK940 was cultured using a fermenter to produce oligosaccharides, and the resulting oligosaccharides with a degree of polymerization greater than 4 were purified by Bio-gel P2 gel permeation column chromatography and then lyophilized. When macrophages were treated with the purified oligosaccharides at concentrations of 0.1–10 mg/mL, no cytotoxicity towards the macrophages was observed. However, nitric oxide production levels were similar to those following treatment with 1 μg/mL lipopolysaccharide. The mRNA expression levels of tumor necrosis factor-α, interleukin-1β, interleukin-6, and inducible nitric oxide synthase were all also increased in a dose-dependent manner following treatment with the oligosaccharides. These data suggest that oligosaccharides produced by *Leuconostoc lactis* CCK940 could be used as an immune enhancer of macrophages.

## 1. Introduction

Lactic acid bacteria such as *Leuconostoc*, *Streptococcus*, and *Weissella* spp. can produce oligosaccharides through the action of enzymes such as glucansucrase (EC 2.4.1.5), levansucrase (EC 2.4.1.10), and alternansucrase (EC 2.4.1.140) [1,2,3]. It has been reported that oligosaccharides can be produced by glucansucrase in *Weissella cibaria* RBA12 [3], fructo-oligosaccharides can be synthesized by levansucrase in *Leuconostoc mesenteroides* Lm17 [4], and cellobiose can be synthesized by alternansucrase from *Leu. mesenteroides* NRRL B-23192 [5]. The enzymatic reaction used for the production of oligosaccharides is affected by the type and concentration of substrate, pH, reaction time, temperature, ratio of acceptor and donor molecules, cell mass, and various inorganic salts [6,7,8]. Among these, the most important factor that affects enzyme reactions is the ratio of acceptor to donor molecules. The acceptor and donor reaction catalyzed by glucansucrase occurs through the transfer of the D-glycosyl group from sucrose, which is used as the donor molecule, to an acceptor sugar to form oligosaccharides [9]. Various molecules can be used as acceptor molecules, including maltose, isomaltose, and nigerose, and it has been reported that the oligosaccharide production efficiency is highest when maltose is used as the acceptor [9,10]. Previous study has isolated *Leuconostoc lactis* CCK940, a lactic acid bacteria which showed high glucansucrase activity, and thereby, producing oligosaccharide with high efficiency, and optimized the reaction conditions, such as pH of the reaction mixture for glucansucrase from *Leu. lactis* CCK940 using maltose as the acceptor molecule [11]. On the basis of these prior data, in the present study, the concentration of acceptor and donor molecules and the reaction temperature were selected as the most important factors for oligosaccharide production, and a response surface methodology (RSM) was used to optimize these three factors. RSM is a statistical experimental design method that can be used to construct mathematical combinations of various variables, in order to select the main parameters among the various parameters in an experiment for increasing the reliability of the experimental result [12,13].

There have been numerous reports on the immunoregulatory effects of oligosaccharides. Xylo-oligosaccharides have been reported to induce the expression of tumor necrosis factor alpha (*TNF-α*), nitric oxide (NO), interleukin (IL)-1β, and *IL-6* in RAW264.7 cells [14]. Alginate oligosaccharides have also been shown to induce NO production and inducible nitric oxide synthase (*iNOS*) expression in a dose- and time dependent manner, and to stimulate the production of reactive oxygen species (ROS) and *TNF-α* [15]. To date, few studies have evaluated the immunostimulatory effects of oligosaccharides produced by lactic acid bacteria.

In the present study, we used RSM′s central composite design (CCD), a statistical tool, to optimize the culture temperature and the concentrations of sucrose and maltose for the production of oligosaccharides from *Leu. lactis* CCK940. The immunostimulatory effect of these oligosaccharides on RAW264.7 macrophage cells was also demonstrated.

## 2. Results and Discussion

### 2.1. Optimization of Oligosaccharide Production Using a Statistical Technique

In order to optimize the conditions for oligosaccharide production by *Leu. lactis* CCK940 through its glucansucrase activity, the RSM methodology was used with CCD. As previously reported, this bacterial strain has been found to use sucrose as a donor and maltose as an acceptor to produce oligosaccharides when cultured in medium at pH 6.0 [11]. Based on the above conditions, the culture temperature and the concentrations of sucrose and maltose, with five levels for each condition, were optimized. As shown in Table 1, it was confirmed that the amount of oligosaccharides produced varied according to the variable level. When *X*_1_ (temperature) was 35 °C, *X*_2_ (sucrose concentration) was 7.5%, and *X*_3_ (maltose concentration) was 7.5%, the amount of oligosaccharide was the lowest, being 97.8 as peak area, whereas when *X*_1_, *X*_2_, and *X*_3_ were 30 °C, 12.5%, and 7.5%, respectively, the highest amount of oligosaccharide was produced, being 312.6.

The appropriateness of the experimental model used in this study was confirmed assuming that a significance of 0.000 was suitable for the model, whereas in the lack of fit test the significance was 0.104 (Table 2).

The coefficients of regression were calculated and the following regression equation was obtained.

*Y* = −3250 + 259.2*X*_1_ − 40.4*X*_2_ − 57.0*X*_3_ − 5.051*X*_1_^2^ − 1.904*X*_2_^2^ − 0.749*X*_3_^2^ + 2.76*X*_1_*X*_2_ + 2.41*X*_1_*X*_3_ − 0.13*X*_2_*X*_3_ (1)

where, *Y* is oligosaccharide production, *X*_1_ is the culture temperature, *X*_2_ is the sucrose concentration, and *X*_3_ is the maltose concentration.

**Figure 1:**
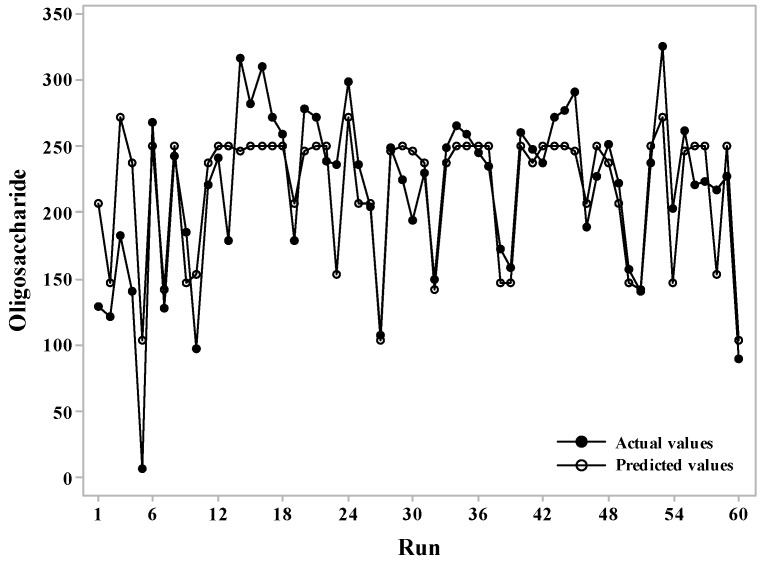
Time series analysis comparing actual and predicted oligosaccharide production.

The analysis of variance of the reaction revealed a coefficient of determination (R-squared) was 0.6599. A time series plot was used to confirm the significance of the actual experimental value versus the predicted values obtained through the CCD, as shown in Figure 1. The differences between the two values were small, indicating that the predicted values and the experimental values were similar using this experimental design.

**Figure 2:**
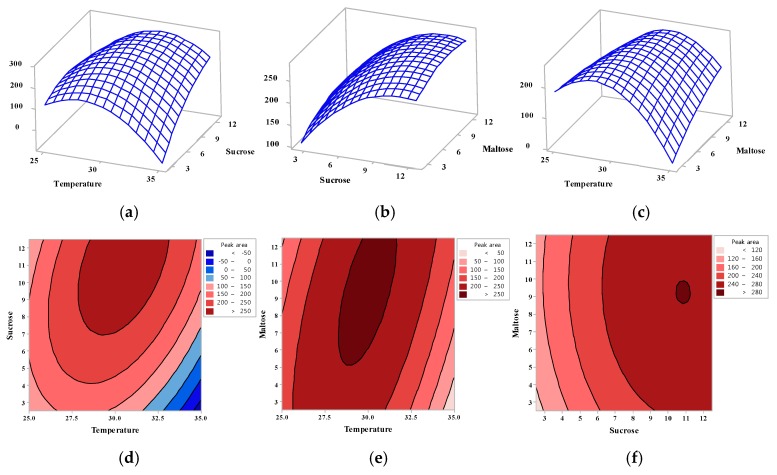
Three-dimensional response surface plots (**a**–**c**) and contour plots (**d**–**f**) showing the optimization of oligosaccharide production by assessing the interactions between temperature, sucrose concentration, and maltose concentration. (**a**) Response surface plot showing the effect of temperature (*X*_1_) and sucrose concentration (*X*_2_) on oligosaccharide production (**b**) Response surface plot showing the effect of temperature (*X*_1_) and maltose concentration (*X*_3_) on oligosaccharide production (**c**) Response surface plot showing the effect of sucrose concentration (*X*_2_) and maltose concentration (*X*_3_) on oligosaccharide production (**d**) Contour plot showing the effect of temperature (*X*_1_) and sucrose concentration (*X*_2_) on oligosaccharide production (**e**) Contour plot showing the effect of temperature (*X*_1_) and maltose concentration (*X*_3_) on oligosaccharide production (**f**) Contour plot showing the effect of sucrose concentration (*X*_2_) and maltose concentration (*X*_3_) on oligosaccharide production.

Figure 2 shows the effect of each variable on the formation of oligosaccharides as three-dimensional plots (Figure 2a–c) and contour lines (Figure 2d–f). As a result, the optimal values for the factors affecting the production of oligosaccharides could be predicted by CCD, with the relative peak area for oligosaccharide production being predicted to be 274.50, using a culture temperature of 30 °C, and concentrations of sucrose and maltose of 9.6% and 7.4%, respectively.

Majumder and Goyal optimized the culture conditions of the *Leu. dextranicum* NRRL B-1146 strain using RSM, and the optimal concentration of Tween 80, sucrose, and K_2_HPO_4_ were determined to be 0.55% (*w*/*v*), 5.6% (*w*/*v*), and 1% (*w*/*v*), respectively, resulting in a yield of glucosyltransferase of 6.40 U/mL [16]. Using RSM, Kanimozhi et al. [7] have also reported that the yield for dextran production using *W. cibaria* NITCSK4 was highest when the sucrose concentration was 15.78%, the yeast extract was 1.27%, and K_2_HPO_4_ was 1.25% at 26 °C. The optimum concentration of sucrose in the present study was 9.6% (*w*/*v*), which was lower than the results of Kanimozhi et al. [7].

It is well-known that the type of oligosaccharide produced by lactic acid bacteria can differ, as can the optimum concentration of sucrose [7,16]. However, few RSM studies have reported the optimization of maltose concentration (as an acceptor) in the production of oligosaccharides.

**Figure 3:**
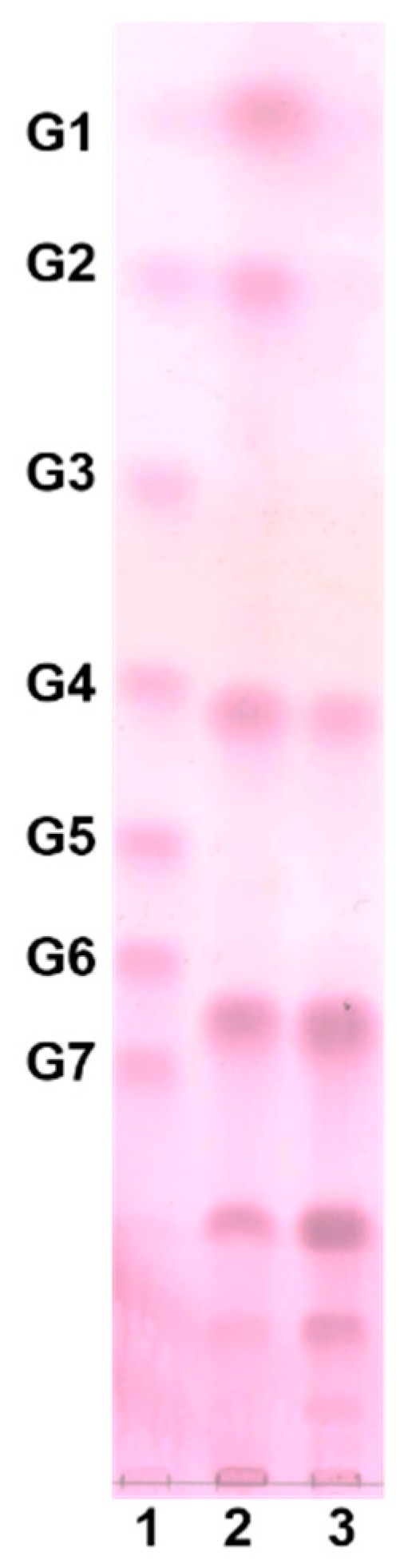
TLC chromatogram of oligosaccharides before and after purification. (1: glucose polymers, G1–G7; 2: before purification; 3: after purification by Bio-gel P2).

Oligosaccharide production was carried out using a jar fermenter (working volume 2 L) at a culture temperature of 30 °C, a sucrose concentration of 9.6%, and a maltose concentration of 7.4%, in order to verify the optimized culture condition determined by CCD. When cultured using the optimized conditions, the oligosaccharide production had a peak area of 241.09, which was slightly different from the predicted value of 274.50. The resulting oligosaccharides were purified using Bio-gel P2, and analyzed by thin layer chromatogram (TLC) to obtain oligosaccharide fractions with a degree of polymerization (DP) of four to nine (Figure 3). The purified oligosaccharide was identified as gluco-oligosaccharide, which consisted of glucose only [17]; however, the type of glycosidic linkage among glucose units need to be identified. The collected fractions were lyophilized and used to examine their immunostimulating effect.

### 2.2. Effect of Oligosaccharides on RAW264.7 Cell Viability and NO Secretion

**Figure 4:**
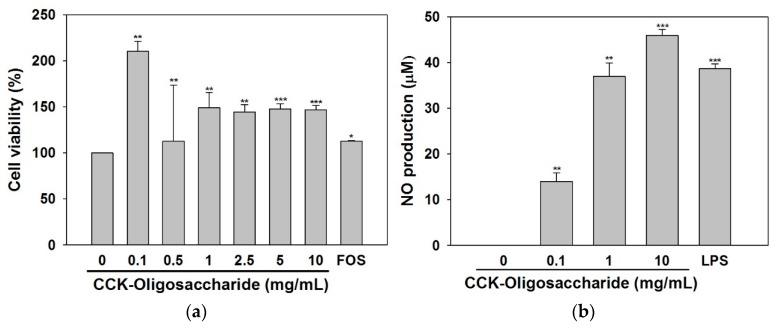
(**a**) Effects of purified oligosaccharides and 1 mg/mL of fructo-oligosaccharide (FOS) on the viability of RAW264.7 murine macrophages (5 × 10^4^ cells/well). (**b**) Effects of the purified oligosaccharides on NO production in RAW264.7 murine macrophages (5 × 10^5^ cells/well). Data are presented as means ± standard deviation of three independent experiments. * *p* < 0.05, ** *p* < 0.01, and *** *p* < 0.001 vs. non-treated oligosaccharides.

Since macrophages play an important role as antigens presenting microbicidal cells and can play a tumoricidal role in cellular or humoral immunity, they are often used to measure the immunological activity of a particular substance [18]. In the present study, the viability of RAW264.7 cells was determined in the presence of different concentrations of purified oligosaccharides (Figure 4a). The purified oligosaccharides stimulated the proliferation of macrophages, without any cytotoxicity at all concentrations used in this study, indicating a greater proliferative capacity than fructo-oligosaccharides.

In previous studies on the cytotoxicity of oligosaccharides, xylo-oligosaccharide was found to be non-toxic towards RAW264.7 cells at concentrations of 0.1–100 μg/mL, and polysaccharides isolated from *Cordyceps sinensis* were found to be non-toxic towards macrophages at concentrations of 0.1–3.0 μg/mL [14,19]. Up to a concentration of 10 mg/mL, the purified oligosaccharides were not toxic to RAW264.7 cells (Figure 4a), and the oligosaccharide concentrations of 0.1, 1.0, and 10 mg/mL were used for further experiments.

Activation of macrophages increases the production of cytokines and NO, and promotes the immune response. NO is an inorganic vitreous substance produced from L-arginine by the action of NO synthase, and a high concentration of NO promotes vital immune responses, as well as vasodilation, and increases phagocytosis as part of the nonspecific host defense mechanism [20].

The production of NO by RAW264.7 cells treated with lipopolysaccharide (LPS) or the purified oligosaccharides is shown in Figure 4b. When the cells were treated with LPS only, NO production was measured to be 38.7 μM. NO production was also stimulated by the purified oligosaccharides in a dose-dependent manner with a maximal level of 45.9 μM NO following treatment with 10 mg/mL oligosaccharides.

A previous study showed that treatment of macrophage cells with an enzymatically hydrolyzed unsaturated guluronate oligosaccharide (0.1, 0.5, and 1 mg/mL) resulted in a dose-dependent increase in NO [15], and similarly NO production was also increased in a concentration-dependent manner when macrophages were treated with 0.1, 0.6, 3, or 15 μg/mL of purified glucogalactomannan from *C. sinensis* [19]. Therefore, the present study is in agreement with several other studies that have shown that some oligosaccharides or polysaccharides have immune-stimulating effects, and can stimulate macrophages to induce NO production, suggesting they could be highly useful as immune active substances.

### 2.3. Effect of Oligosaccharides on Cytokine and *iNOS* Secretion

The activation of macrophages leads to production of inflammatory mediators such as *TNF-α*, *IL-1β*, etc., and these cytokines act to prevent the invasion of non-specific pathogens, such as bacteria and parasites, as part of the innate immune response where they play an important role in phagocytosis [21].

*TNF-α* is an important multifunctional cytokine that is involved in host defense against pathogens, and can inhibit tumor cell differentiation through antibody-dependent or macrophage-mediated cytotoxicity. This cytokine acts on monocytes and macrophages and promotes immune cell function [22,23].

In addition, *IL-1β*, a pro-inflammatory cytokine, induces various immunoregulatory functions such as cell differentiation, proliferation, and apoptosis [24], whereas *IL-6* acts as an important mediator of the acute inflammatory response and increases phagocytosis and complement production. It can also function as a growth factor to promote the differentiation of endothelial cells, neuronal cells, keratinocytes, B cells, and T cells [25].

**Figure 5:**
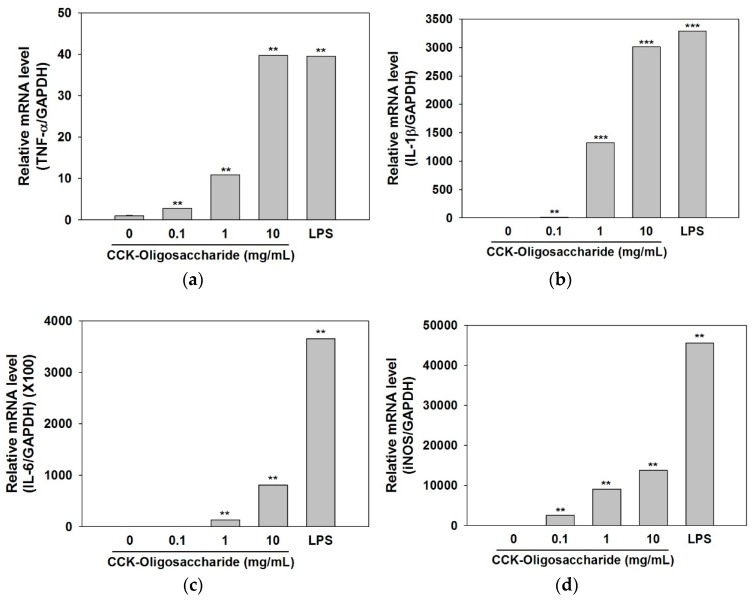
Effects of the purified oligosaccharides on the mRNA expression levels of *TNF-α* (**a**), *IL-1β* (**b**), *IL-6* (**c**), and *iNOS* (**d**) in RAW264.7 murine macrophages. Data are presented as the means ± standard deviation of three independent experiments. *p* < 0.05, ** *p* < 0.01, and *** *p* < 0.001 vs. non-treated oligosaccharides.

In order to analyze the immunostimulatory effect of the purified oligosaccharides produced in this study, the mRNA expression levels of *TNF-α*, *IL-1β*, and *IL-6* were assessed using real-time PCR (Figure 5a–c). The mRNA expression levels of *TNF-α* and *IL-1β* were increased in a dose-dependent manner following treatment of macrophages with the purified oligosaccharides, with similar mRNA expression level observed in cells treated with the maximal concentration of 10 mg/mL oligosaccharides or with 1 mg/mL LPS (Figure 5a,b). When cells were treated with oligosaccharides at the maximal concentration of 10 mg/mL, the mRNA expression level of *IL-6* was 4.5 times lower than that with LPS only, although the increases in *IL-6* were still dose-dependent (Figure 5c).

These results suggest that the oligosaccharides produced from *Leu. lactis* CCK940 directly activate the innate immune system, such as macrophages, which is the first step in an immune response, to increase the production of cytokines such as *TNF-α*, *IL-1β*, and *IL-6*.

Activated macrophages also produce oxygen radicals along with NO, which have antimicrobial activity. NO is a biologically active molecule with high reactivity, which is produced from L-arginine by NOS (nitric oxide synthase) and plays an important role as a secondary signal transducer in cells [26]. Inducible NOS (*iNOS*) is increased following stimulation with LPS, interferon-γ, IL-1, and *TNF-α*, and the resulting high concentrations of nitric oxide produced in vivo promotes vascular dilation as well as various immune responses [27,28].

As shown in Figure 5d, oligosaccharides were found to increase the mRNA expression levels of *iNOS* in macrophages in a dose-dependent manner; this in turn would increase the production of nitric oxide by the macrophages. When cells were treated with oligosaccharides at concentrations of 1 and 10 mg/mL, the *iNOS* expression levels were 5 and 3.3 times lower, respectively, than that following treatment with LPS alone. LPS is not clinically used as an immunostimulating agent due to its uncontrollably strong immunostimulant activity [29]; however, the oligosaccharides in the present study might be useful as immunostimulating agents because they can induce the expression of cytokines in a concentration-dependent manner, and can thereby control their immunostimulating activity. These results and the previous study [17] indicated that the immunostimulant activity of oligosaccharide produced in this study is due to the activation of MAPK signaling pathway in macrophage cells; however, the fully explained mechanism should be elucidated by further study.

## 3. Materials and Methods

### 3.1. Bacterial Strains and Culture Conditions

The strain *Leu. lactis* CCK940 (GenBank accession number NZ_NQLF00000000) used in this study was isolated from home-made kimchi purchased from a traditional Korean market [11]. This strain was cultured using Lactobacilli MRS broth (BD, Franklin Lakes, NJ, USA) at 30 °C for 20 h.

### 3.2. Response Surface Methodology Using Central Composite Design

In order to optimize oligosaccharide production, independent variables of the reaction temperature (25, 27.5, 30, 32.5, 35 °C), sucrose concentration (2.5, 5.0, 7.5, 10.0, 12.5%, *w*/*v*), maltose concentration (2.5, 5.0, 7.5, 10.0, 12.5%, *w*/*v*), and five variables levels (−2, −1, 0, 1, 2) were set according to the central composite design and supplementary experiments were performed by repeating the experiment three times for each of the 20 experimental conditions. The high-performance anion-exchange chromatography peak areas of the oligosaccharides produced were obtained by pulsed amperometric detection (HPAEC-PAD), and were used as dependent variables affected by these factor variables. A response surface regression analysis and the characteristics of dependent variables with independent variables were analyzed using Minitab 17.2.1 (Minitab Pty Ltd., Sydney, Australia).

### 3.3. Analysis of Oligosaccharides by HPAEC-PAD

The oligosaccharides produced by *Leu. lactis* CCK940 were analyzed by HPAEC-PAD (DX 500 Chromatography System, Dionex, Sunnyvale, CA, USA). The *Leu. lactis* CCK940 culture was centrifuged at 27,237× *g* for 1 min and the supernatant was analyzed by HPAEC-PAD (Dionex) equipped with a CarboPac PA-100 column (4 × 250 mm, Dionex) and a CarboPac PA-100 guard column (4 × 50 mm, Dionex). The flow rate was 1.0 mL/min, and the mobile phase used for oligosaccharide separation was 150 mM sodium hydroxide for the first 20 min, after which 600 mM sodium acetate (in 150 mM sodium hydroxide) was applied with a gradient of 60:40 to 0:100 from 20 to 25 min, and 150 mM sodium hydroxide was used from 25 to 40 min (sodium hydroxide, Fisher Scientific, Hampton, NH, USA; sodium acetate, Sigma-Aldrich, St. Louis, MO, USA). Ten microliters of each sample was injected for each analysis.

### 3.4. Purification of Oligosaccharides

Batch cultures were conducted using a working volume of 2 L in a 3-L jar fermenter (Fermentec, Cheongju, Korea) according to the optimum oligosaccharide production conditions determined using the RSM.

The culture was centrifuged at 9820× *g* for 15 min (Beckman Coulter, Brea, CA, USA) and the supernatant was concentrated under reduced pressure at 60 °C. The concentrated supernatant was then loaded onto Bio-Gel P2 (fine mesh) in a Glass Econo-Column (1.5 × 120 cm) and the oligosaccharides were separated by gel permeation chromatography (GPC) (Bio-Rad, Hercules, CA, USA). The flow rate was 0.5 mL/min, and the eluents were fractionated using a fraction collector (Gilson Inc., Middleton, WI, USA) with 5 mL per tube. The fractions were analyzed by TLC and the fractions containing oligosaccharides were collected and lyophilized (SunilEyela, Seongnam, Korea) to check their immunological effects. Silica gel 60 F254 (Merck, Darmtadt, Germany) was used for TLC, and the samples were developed twice using 2:5:1.5 nitromethane (Sigma-Aldrich): *n*-propyl alcohol (Samchun, Seoul, Korea):water. The developed TLC plate was dipped in 0.3 %(*w*/*v*) *N*-(1-naphtyl) ethylenediamine dihydrochloride (Sigma-Aldrich) and 5%(*v*/*v*) sulfuric acid (Duksan, Gyeonggi, Korea) in methanol (CARLO ERBA Reagents S.A.A., Val de Reuil, France) and then baked at 121 °C for 5 min. Glucose polymer (DP 1-7) purchased from Carbosynth Co. (Berkshire, UK) was used as a standard sugar.

### 3.5. Cell-Based Immunostimulatory Effect Test

#### 3.5.1. Cell Culture

RAW264.7 cells, a murine macrophage cell line, were cultured in DMEM (Gibco, Grand Island, NY, USA) supplemented with 10% bovine serum (Gibco) and 1% penicillin-streptomycin (Gibco) at 37 °C in a 5% CO_2_ incubator.

#### 3.5.2. Cell Viability

The viability of macrophages was determined using Cell Counting Kit-8 (EZ-CYTOX, DAEILLAB Service Co., Ltd., Seoul, Korea). RAW264.7 macrophages were seeded in a 96-well plate at 5 × 10^4^ cells/well and pre-incubated for 20 h. CCK-oligosaccharide was added at a concentration of 0.1, 0.5, 1.0, 2.5, 5.0, and 10.0 mg/mL, and cultured at 37 °C in a 5% CO_2_ incubator. As a positive control, fructo-oligosaccharide was used at concentration of 1 mg/mL. After incubation, the absorbance was measured at 450 nm (Epoch microplate reader, Biotek Instruments, Inc., Winooski, VT, USA).

#### 3.5.3. NO Assay

Following treatment of RAW264.7 macrophages with different concentrations of oligosaccharides, the amount of NO released into the medium was quantified using the Griess method. The cells were seeded in a 24-well plate at 5 × 10^5^ cells/well for 20 h, and then treated with oligosaccharides at concentrations of 0.1, 1.0, and 10.0 mg/mL. The cells were also treated with 1 μg/mL LPS (Sigma-Aldrich) as a control. Cells were further cultured for 24 h, after which 100 μL of the culture supernatant was collected and mixed with 100 μL of Griess reagent and allowed to react at room temperature for 15 min, following which the absorbance was measured at 540 nm (Epoch microplate reader, Biotek Instruments, Inc.). Griess reagent A was 0.1% *N*-1-naphthyl ethylene diamine and reagent B was 5% phosphoric acid with 1% sulfanilamide. These solutions were mixed at a ratio of 1:1 immediately before use (Sigma-Aldrich). A standard curve was prepared by diluting sodium nitrite (Sigma-Aldrich) stepwise to give solutions of 0–250 μM.

#### 3.5.4. Expression of Cytokines

The expression levels of cytokines were measured in order to confirm the immunostimulating effect of the oligosaccharides on RAW264.7 cells. Cells were cultured in a 6-well plate at 2 × 10^6^ cells/well, and treated with oligosaccharides at 0.1, 1.0, and 10.0 mg/mL, or treated with 1 μg/mL LPS as a control, and then incubated at 37 °C in a 5% CO_2_ incubator. RNA was isolated using a QIAGEN RNeasy RNA isolation kit (QIAGEN, Hilden, Germany) from the cultured cells. Transcriptor First Strand cDNA Synthesis Kit (Roche, Basel, Switzerland) was used to synthesize cDNA from the isolated RNA, and the synthesized cDNA was used for real-time PCR (LightCycler96, Roche) using the FastStart Essential DNA Green Master Kit (Roche). The cytokine genes amplified in this study were *TNF-α*, *IL-1β*, *IL-6*, and *iNOS*. Glyceraldehyde 3-phosphate dehydrogenase (*GAPDH*) was used as an endogenous control gene, and relative expression levels of these cytokine mRNA compared with the levels of the *GAPDH* mRNA, were analyzed using the 2^−ddCT^ method [16]. The sequences of the primers used in this study are listed in Table 3.

#### 3.5.5. Statistical Analysis

Data are expressed as mean ± standard deviation (SD) from triplicate experiments. Statistical analyses were performed using SPSS 23 (SPSS Inc., Chicago, IL, USA). Statistical significance between groups was determined by a paired *t*-test for repeated measures. Data with *p* < 0.05, *p* < 0.01, and *p* < 0.001 were considered statistically significant.

## 4. Conclusions

In this study, the factors affecting the acceptor-donor reaction were optimized using RSM to for the efficient production of immune active oligosaccharides from a lactic acid bacteria. RSM data enabled us to produce oligosaccharide with high efficiency. This study is meaningful in that the production of gluco-oligosaccharide from *Leu. lactis* has never been reported. When macrophage cells were treated with the purified oligosaccharides produced in this study, the expression levels of cytokines such as *TNF-α*, IL-1, *IL-6*, and *iNOS* were increased. These molecules are involved in NO production and immunity enhancement. In addition, the purified oligosaccharides stimulated cell proliferation. These results imply that immune mediators would be produced as a result of activation of immune cells by these oligosaccharides, thereby increasing the nonspecific immune response, and thus, could play an important role in the natural immune response.

## Figures and Tables

**Table 1:** Central composite design for independent variables and the resulting oligosaccharide production.

Run	Coded Variable Levels			Oligosaccharide Production (Relative Peak Area)
	Temperature, °C (*X*_1_)	Sucrose, % (*X*_2_)	Maltose, % (*X*_3_)	
1	27.5 (−1)	5.0 (−1)	10.0 (+1)	212.9 ± 13.1 *
2	32.5 (+1)	5.0 (−1)	5.0 (−1)	145.1 ± 20.5 **
3	30 (0)	12.5 (+2)	7.5 (0)	312.6 ± 19.4 *
4	27.5 (−1)	10.0 (+1)	10.0 (+1)	248.9 ± 1.1 **
5	35 (+2)	7.5 (0)	7.5 (0)	97.8 ± 12.8
6	30 (0)	7.5 (0)	7.5 (0)	270.3 ± 1.9 ***
7	30 (0)	2.5 (−2)	7.5 (0)	139.0 ± 10.7 **
8	30 (0)	7.5 (0)	7.5 (0)	248.1 ± 26.7 **
9	32.5 (+1)	5.0 (−1)	10.0 (+1)	186.8 ± 14.8 **
10	25 (−2)	7.5 (0)	7.5 (0)	227.0 ± 13.5 *
11	27.5 (−1)	10.0 (+1)	5.0 (−1)	234.1 ± 15.7 **
12	30 (0)	7.5 (0)	7.5 (0)	244.7 ± 19.5 **
13	30 (0)	7.5 (0)	2.5 (−2)	231.1 ± 10.9 *
14	32.5 (+1)	10.0 (+1)	10.0 (+1)	304.1 ± 18.6 *
15	30 (0)	7.5 (0)	7.5 (0)	259.6 ± 23.0 **
16	30 (0)	7.5 (0)	7.5 (0)	233.5 ± 17.0 *
17	30 (0)	7.5 (0)	7.5 (0)	245.0 ± 23.4 **
18	30 (0)	7.5 (0)	12.5 (+2)	252.3 ± 12.9 **
19	27.5 (−1)	5.0 (−1)	5.0 (−1)	183.8 ± 7.6 *
20	32.5 (+1)	10.0 (+1)	5.0 (−1)	270.3 ± 12.1 *

Data are presented as means ± standard deviation of three independent experiments. (* *p* < 0.05, ** *p* < 0.01, and *** *p* < 0.001).

**Table 2:** Analysis of variance of the experimental results using a central composite design.

Variables	DF	Adj SS	Adj MS	F-Value	*p*-Value
Model	9	151,743	16,860.3	10.78	0.000
Linear	3	61,162	20,387.5	13.03	0.000
Quadratic	3	77,970	25,990.1	16.61	0.000
Cross-product	3	12,610	4203.4	2.69	0.056
*X*_1_	1	7750	7750.0	4.95	0.031
*X*_2_	1	50,631	50,631.1	32.37	0.000
*X*_3_	1	2781	2781.3	1.78	0.188
*X*_1_^2^	1	75,158	75,157.8	48.04	0.000
*X*_2_^2^	1	10,678	10,678.1	6.83	0.012
*X*_3_^2^	1	1653	1653.5	1.06	0.309
*X*_1_ *X*_2_	1	7159	7159.0	4.58	0.037
*X*_1_ *X*_3_	1	5436	5436.4	3.48	0.068
*X*_2_ *X*_3_	1	15	15.0	0.01	0.922
Residual	50	78,217	1564.3	-	-
Lack of fit	5	13,947	2789.5	1.95	0.104
Pure error	45	64,270	1428.2	-	-
Cor Total	59	229,960			

**Table 3:** Cytokine primer sequences.

Gene		Sequence
*GAPDH*	Forward	5′―ATC CCA TCA CCA TCT TCC AG―3′
	Reverse	5′―CCT GCT TCA CCA CCT TCT TG―3′
*TNF-α*	Forward	5′―ATG AGC ACA GAA AGC ATG ATC CG―3′
	Reverse	5′―CCA AAG TAG ACC TGC CCG GAC TC―3′
*IL-1β*	Forward	5′―ATG GCA ACT GTT CCT GAA CTC AAC T―3′
	Reverse	5′―CAG GAC AGG TAT AGA TTC TTT CCT T―3′
*IL-6*	Forward	5′―CAA GAG ACT TCC ATC CAG TTG C―3′
	Reverse	5′―TTG CCG AGT TCT CAA AGT GAC―3′
*iNOS*	Forward	5′―AAT GGC AAC ATC AGG TCG GCC ATC ACT―3′
	Reverse	5′―GCT GTG TGT CAC AGA AGT CTC GAA CTC―3′
